# Caliper measurement to improve assessment of neck lumps: comment 1

**DOI:** 10.1308/003588413X13511609956534

**Published:** 2013-01

**Authors:** P Brennan

**Affiliations:** Portsmouth Hospitals NHS Trust,UK

## Abstract

Although this comment was published originally in the September 2012 issue of the *Annals*, we omitted to publish the author’s response alongside it. We include Mr Wasson’s response below and apologise for any inconvenience caused.

**Comment on**

**Wasson J, Amonoo-Kuofi K, Scrivens J, Pfleiderer A** Caliper measurement to improve clinical assessment of palpable neck lumps. *Ann R Coll Surg Engl* 2012; **94**: 256–260

doi 10.1308/003588412X13171221499784

I read the above paper with interest. It is certainly an easy-to-use technique to monitor the size of readily palpable lumps that are seen by head and neck specialists and it would seem to increase the accuracy of clinical measurement. However, I was concerned that the authors stated that as a result of increasing numbers of referrals, not all new patients with a palpable neck lump will go on to have ultrasonography and that calipers can improve clinical assessment, particularly when an ultrasonography machine is not available.

They also mentioned that all patients with a lump greater than 9mm in their unit will go on to have ultrasonography. The authors make no mention of what the upper limits of normal size for lymph nodes are in various levels of the neck; these vary depending on site. For example, a 15mm jugulodigastric node with a short axis on ultrasonography less than 9mm may well be reactive while a similar size node in the submental area is almost always pathological and requires fine needle aspiration to exclude malignancy.^1^

The additional advantage of ultrasonography is that it can confirm a reactive node at the first visit not only by short axis measurement but also by demonstrating normal hilar architecture and blood flow using colour flow Doppler. None of these assessments can be made using clinical examination or calipers and, consequently, patients having clinical assessment alone will undoubtedly be followed up in a review clinic instead of being reassured and discharged.

Therefore, perversely, not having access to ultrasonography may result in additional clinic visits as well as potentially delaying a malignant diagnosis irrespective of better accuracy in determining the lymph node size using calipers. In addition to diagnosing metastatic disease, lymphoma nodes (which in certain subtypes can remain small for some time) often have readily visualised ultrasonography appearances and rapid diagnosis can be made using ultrasonography guided tru-cut biopsy.^2^

Finally, the authors make no mention of oral and maxillofacial surgeons managing neck lumps. In many units in the UK, both otolaryngologists and oral and maxillofacial surgeons work together to provide a high quality neck lump service with a head and neck radiologist; many patients can be discharged at the first visit following clinical assessment and ultrasonography.
